# Mouse RC/BTB2, a Member of the RCC1 Superfamily, Localizes to Spermatid Acrosomal Vesicles

**DOI:** 10.1371/journal.pone.0039846

**Published:** 2012-06-29

**Authors:** Jiannan Wang, Maria E. Teves, Xuening Shen, David R. Nagarkatti-Gude, Rex A. Hess, Scott C. Henderson, Jerome F. Strauss, Zhibing Zhang

**Affiliations:** 1 Department of Obstetrics & Gynecology, Virginia Commonwealth University, Richmond, Virginia, United States of America; 2 Department of Biochemistry & Molecular Biology, Virginia Commonwealth University, Richmond, Virginia, United States of America; 3 Department of Anatomy & Neurobiology, Virginia Commonwealth University, Richmond, Virginia, United States of America; 4 Department of Comparative Biosciences, College of Veterinary Medicine, University of Illinois, Urbana, Illinois, United States of America; National Cancer Institute, United States of America

## Abstract

Mouse RC/BTB2 is an unstudied protein of the RCC1 (Regulator of Chromosome Condensation) superfamily. Because of the significant remodeling of chromatin that occurs during spermiogenesis, we characterized the expression and localization of mouse RC/BTB2 in the testis and male germ cells. The *Rc/btb2* gene yields two major transcripts: 2.3 kb *Rc/btb2-s*, present in most somatic tissues examined; and 2.5 kb *Rc/btb2-t,* which contains a unique non-translated exon in its 5′-UTR that is only detected in the testis. During the first wave of spermatogenesis, *Rc/btb2-t* mRNA is expressed from day 8 after birth, reaching highest levels of expression at day 30 after birth. The full-length protein contains three RCC1 domains in the N-terminus, and a BTB domain in the C-terminus. In the testis, the protein is detectable from day 12, but is progressively up-regulated to day 30 and day 42 after birth. In spermatids, some of the protein co-localizes with acrosomal markers sp56 and peanut lectin, indicating that it is an acrosomal protein. A GFP-tagged RCC1 domain is present throughout the cytoplasm of transfected CHO cells. However, both GFP-tagged, full-length RC/BTB2 and a GFP-tagged BTB domain localize to vesicles in close proximity to the nuclear membrane, suggesting that the BTB domain might play a role in mediating full-length RC/BTB2 localization. Since RCC1 domains associate with Ran, a small GTPase that regulates molecular trafficking, it is possible that RC/BTB2 plays a role in transporting proteins during acrosome formation.

## Introduction

Spermatogenesis can be divided into three stages: spermatogonial mitosis, meiosis of spermatocytes, and spermiogenesis. In the first stage, spermatogonia proliferate by mitosis, from which, the last mitotic division of each spermatogonium yields two primary spermatocytes [1]. The meiotic stage comprises two successive divisions. Spermiogenesis occurs after the second meiotic division; the haploid round spermatids differentiate into the species-specific shaped spermatozoon. During this period, major cell restructuring occurs. The acrosome, a vesicle containing a group of proteolytic enzymes, develops [2]; the nucleus is transformed from a nucleosome-containing structure into the tightly compacted nucleus [3]; and a tail is formed [4]. As a result of these structural changes during spermiogenesis, many testis-specific proteins must be synthesized; these proteins are involved in the regulation of chromosomal packaging, acrosome biogenesis, and flagella formation [5].

A number of genes have been identified to be essential for normal spermiogenesis by gene targeting [6]. One of them is mouse sperm-associated antigen 16 (*Spag16*) gene. Mouse *Spag16* is a bi-functional gene that encodes full-length 71 kDa SPAG16L, which is assembled into sperm flagella and regulates sperm motility [7], and 35 kDa SPAG16S, which is also localized in the nuclei of round spermatids and is essential for spermatogenesis [8]. Both proteins contain seven copies of the WD motif, a conserved domain that mediates protein-protein interactions [9]. Yeast two-hybrid screens conducted using the SPAG16 WD repeats as bait to search for potential binding partners identified SPAG6 [10], TSSK2 [11], and MEIG1 [8], [12]. In the same screen, mouse chromosome condensation 1-like (BC003224), now called RC/BTB2, was identified as another potential binding partner [11].

Mouse RC/BTB2 is a protein that belongs to RCC1 (Regulator of Chromosome Condensation) superfamily. The RCC1 superfamily of proteins is characterized by a 350–500 residue domain, known as the RCC1-like domain or RLD, that was first reported in 1987 in the Regulator of Chromosome Condensation 1 [13]. These RCC1 superfamily proteins can be divided into five subgroups based on structural criteria: (1) the RCC1 subgroup (including those proteins whose RLD spans almost the whole length of the protein), (2) the HERC subgroup (proteins containing RLD and HECT domains), (3) the RCBTB subgroup (proteins containing RLD and BTB domains), (4) the kinase subgroup (proteins containing RLD and kinase domains), and (5) the miscellaneous subgroup (encompassing those proteins that do not fit into any of the previous categories). They perform many different functions, including guanine nucleotide exchange on small GTP-binding proteins, enzyme inhibition or interaction with proteins and lipids [14]–[16].

RC/BTB2 belongs to the RCBTB subgroup. Besides the RCC1 domain, proteins in this family also contain a Bric-a-brac, Tramtrack, and Broad Complex (BTB) domain [17]. Three members of the RCBTB subgroup have been identified to date, RC/BTB1 (CLLD7), human RC/BTB2 (CHC1L) [18], [19], and IBtk (inhibitor of Bruton's tyrosine kinase) [20]. The functions of these proteins are not well known. IBtk might play a role in B cell development [20], RC/BTB1 might be a tumor suppressor in chronic lymphocytic leukemia [21]. Even though RC/BTB2 sequences were reported previously [22], [23], and Latil suggested that it might also be a tumor suppressor in human [24], the protein is largely uncharacterized. As RC/BTB2 has the RCC1 domain, it might play a role in nucleocytoplasmic transport, a key process of spermiogenesis during which the somatic histones are replaced by the transition proteins, TNP1 and TNP2, which are, in turn, replaced by the protamine proteins, P1 and P2, toward the end of spermiogenesis [25]. In addition, as RC/BTB2 might be in the SPAG16 complex, we were interested in the potential role of this protein in spermiogenesis. Consequently, we characterized the temporal pattern of gene and protein expression and protein localization during spermatogenesis. Our findings demonstrate a targeting role for the BTB domain in CHO cells, a known mediator of protein-protein interaction. In spermatids, some RC/BTB2 co-localizes with the acrosomal protein sp56, and peanut lectin, another acrosomal marker, indicating that it is, in part, an acrosomal protein. We speculate that RC/BTB2 plays a role in transporting material inside the acrosome during acrosome formation and acrosomal protein condensation during late spermiogenesis.

## Materials and Methods

### Ethics Statement

All rodent work was approved by Virginia Commonwealth University's Institutional Animal Care & Use Committee (protocol permit #AM10297) in concordance with all federal and local regulations regarding the use of non-primate vertebrates in scientific research.

### Identification of the *Rc/btb2* cDNA

The mouse *Rc/btb2* cDNA (BC003224) was identified from a yeast two-hybrid screen using WD repeats of mouse SPAG16 protein as bait [11, Table 1]. The significance of the interaction between RC/BTB2 and the SPAG16 WD repeats is currently not known. It could reflect an affinity of RC/BTB2 for the WD repeat motif, or specific binding to SPAG16L or SPAG16S.

### Northern blot analysis

A mouse multiple tissue RNA blot was purchased from Clontech (Cat No: 636808, Palo Alto, CA). Total RNA was isolated from mouse testes at indicated ages with Trizol (Life Technologies, Inc., Grand Island, N.Y.), 30 µg of these RNAs were separated on a 1% denaturing agarose gel, the RNA was subsequently transferred to an Hybond N+ nylon membrane. The blot was hybridized with the ^32^P-α-dCTP labeled *Rc/btb2* probe generated by PCR using a primer set based on the mouse cDNA sequence. After hybridization, the membranes were exposed to X-ray films overnight or for four days.

### Rapid amplification of cDNA ends (RACE)

RACE experiments were carried out with the Marathon cDNA amplification kit (Clontech) to define the cDNA ends of the *Rc/btb2* mRNAs using mouse testis and heart poly (A)+ RNA. Briefly, primers were designed within the coding sequences of *Rc/btb2* and used together with the Marathon cDNA adaptor primers to generate 5′-RACE or 3′-RACE products from testis and heart cDNAs. The RACE products were cloned into the pCR2.1-TOPO TA vector (Invitrogen, Carlsbad, CA) and subjected to DNA sequence analysis.

### Reverse transcription–polymerase chain reaction (RT-PCR)

Total RNAs from the indicated tissues were reversed transcribed using the Transcritpor first strand cDNA synthesis kit from Roche. RT-PCR was conducted using cDNAs transcribed from these tissues (Heart, Brain, Spleen, Lung, Liver, Smooth Muscle, Kidney, Seminal Vesicle, Testis and Ovary) to examine the expression of the two *Rc/btb2* mRNA isoforms using specific primer sets described in the Table S1. *Gapdh*, a housekeeping gene, was used as the control. The PCR conditions were incubation at 95°C for 4 min followed by 32 cycles of 95°C for 30 s, 60°C for 45 s, and 72°C for 1 min ending at 72°C for 10 min.

### Green fluorescent protein (GFP) fusion constructs

Complementary DNAs containing the 1668 bp full-length (68 to 1735), 1056 bp N-terminus three RCC1 domains (68 to 1123) and 636 bp C terminus BTB domain (1100 to 1735) of mouse *Rc/btb2* cDNA were amplified by RT-PCR. After sequencing, the PCR products were cloned into *Eco*RI/*Bam*HI sites of the pEGFP-C1 vector, creating the full-length RC/BTB2/pEGFP-C1 and truncated RCC1/pEGFP-C1, and BTB/pEGFP-C1 plasmids.

### Cell culture and transient transfection

Chinese hamster ovary (CHO) cells and COS-1 cells were obtained from the American Type Culture Collection (Manassas, VA) and cultured at 37°C in Dulbecco's modified Eagle's medium containing 10% heat-inactivated fetal bovine serum. At 60% confluence, transfection was conducted with the indicated plasmids using FuGENE6 (Roche, Indianapolis, Ind.) following the manufacturer's instructions.

### Immunofluorescence microscopy of transfected mammalian cells

Cells were grown on two chamber slides. Forty-eight hours after transfection, cells were fixed and permeabilized with methanol. After blocking (PBS containing 10% goat serum), the cells were incubated overnight at 4°C with the primary anti-LaminB (sc-6216, Santa Cruz Biotechnology, Santa Cruz, CA, USA) antibody at a 1∶200 dilution. Primary antibody was detected with secondary anti-goat Cy3 (Jackson ImmunoResearch Laboratories, West Grove, PA). DNA was counterstained with DAPI. Images were taken by confocal laser-scanning microscopy (Leica TCS-SP2 AOBS) and processed using Adobe Photoshop 5.0.

### Biochemical fractionation of transfected mammalian cells

Biochemical fractionation was performed essentially as described by Haque [26] using COS-1 cells transfected with the full-length RC/BTB2/pEGFP-C1 plasmid. In brief, cells were dispersed in 20 volumes of ice-cold hypotonic buffer [10 mM Hepes pH 7.4, 2 mM MgCl_2_, 25 mM KCl, 1 mM dithiothreitol (DTT), 1 mM phenylmethylsulfonyl fluoride (PMSF)] containing complete protease inhibitor cocktail (Roche) for 45 min at 4°C. After addition of 0.5 volumes hypotonic buffer containing 24% sucrose, cells were lysed by application of 100 strokes in a Dounce hand homogenizer, nuclei were pelleted at 400g for 10 min at 4°C and the supernatant (S1) stored. Nuclei were washed in hypotonic buffer containing 8% sucrose and pelleted at 400g at 4°C for 10 min. Nuclei were then resuspended in extraction buffer (10 mM Hepes pH 7.4, 2 mM MgCl_2_, 1 mM PMSF) containing 50 mM NaCl and 1% Triton X-100 and incubated for 30 min on ice. The suspension was then centrifuged at 16,000g for 10 min at 4°C and separated into pellet and soluble supernatant (S2) fractions. The pelleted fraction was resuspended in extraction buffer containing 500 mM NaCl and 1% Triton X-100 and incubated on ice for 30 min. Centrifugation resulted in a third supernatant (S3) and an insoluble fraction. The pellet was once more extracted in 7 M urea containing extraction buffer for 30 min and centrifuged to give rise to supernatant (S4) and insoluble pellet (P). All four supernatants as well as the insoluble last fraction (pellet) were analyzed on denaturing polyacrylamide gels [27].

### Generation of Anti-RC/BTB2 Antibodies

Two cDNAs encoding a N-terminal portion (amino acid residues 1–328) and a C-terminal portion (amino acid residues 373–551) were amplified from a *Rc/btb2* cDNA clone with the primers indicated in the Table S1. The cDNAs were inserted into Nhe I/EcoR I sites of the pET28a vector (Novagen). The constructs were transformed into BL-21 (DE3) cells, induced with IPTG (1mM), and the fusion proteins were subsequently purified using Ni-NTA (QIAGEN) as reported previously [28]. The resulting fusion proteins contained His_6_ tags at both the N- and C- termini. The purified recombinant proteins were used to generate polyclonal antisera in rabbits by a commercial organization (Rockland, Gilbertsville, PA). The antibody produced using N-terminal polypeptide is referred to as 7610 (rabbit identification number). The antibody produced using the C-terminal polypeptide is referred to as 13–22.

### Western blotting analysis

Equal amounts of protein (50 μg/lane) were heated to 95°C for 10 min in 4 X sample buffer, loaded onto 10% sodium dodecyl sulfate-polyacrylamide gels, electrophoretically separated, and transferred to polyvinylidene difluoride membranes. The membranes were blocked and then incubated with the indicated antibodies overnight at 4°C. After washing, the blots were incubated with an anti-rabbit, anti-goat or mouse immunoglobulin conjugated to horseradish peroxidase for 1 h at room temperature. The proteins were detected with Super Signal Pico Chemiluminescent or Femto Maximum Sensitive system (Pierce, Rockford, IL). The migrating distance of the RC/BTB2 protein and the protein markers was measured to estimate the molecular weight of the RC/BTB2 protein.

### Enzyme-dissociated testicular cell preparations and immunofluorescence

Enzyme-dissociated testicular cells were prepared using the method described by Xu et al [29]. In brief, testes from an adult wild-type mouse were decapsulated and placed in 5 mL DMEM containing 0.5 mg/mL collagenase IV (Sigma-Aldrich) and 1.0 mg/mL DNase I (Sigma-Aldrich), and was incubated for 30 min at 32°C to dissociate testicular cells, then centrifuged for 5 min at 1000 rpm. Dispersed mixed testicular cells were fixed by 15 min incubation in 4% paraformaldehyde/PBS (containing 4% sucrose) at room temperature, then washed three times with PBS. Prior to plating, cells were re-suspended in 12.5 mL PBS and 100 µL of cell suspension was spread on SuperFrost/Plus microscope slides (Fisher Scientific) and allowed to air-dry. The cells were permeabilized with 1% Triton X-100 for 5 min at 37°C, blocked for 1 h at room temperature with 10% goat serum in PBS. Following overnight incubation with primary antibodies (diluted in blocking medium at a 1∶200 dilution for anti-RC/BTB2 antibodies, and 1∶100 for anti-α-tubulin antibody) at 4°C, slides were washed with PBS and incubated for 1 h at room temperature with Alexa 488-conjugated anti-mouse IgG secondary antibody (1∶500; Jackson ImmunoResearch Laboratories) or Cy3-conjugated anti-rabbit IgG secondary antibody (1∶5000; Jackson ImmunoResearch Laboratories). Following secondary antibody incubation, slides were washed in PBS and sealed using VectaMount with DAPI (Vector Laboratories, Burlingame, CA). Some cells were double-stained with an acrosome marker, peanut-lectin (Invitrogen, Cat number: L21409) [30] or anti-ZP3R (also called sp56) antibody [31]. Briefly, after the cells were stained with secondary antibody, the cells were washed three times in PBS, and incubated with peanut-lectin (20 µg/ml final concentration) at room temperature for 15 min, washed again three times in PBS, mounted with VectaMount, and sealed with a cover slip. Images were captured by confocal laser-scanning microscopy as before.

### Immunolocalization of RC/BTB2 in mouse testis

Testes from wild-type adult males were fixed with 4% paraformaldehyde in 0.1 M PBS (pH 7.4), and 5 μm paraffin sections were made. C-terminus 13–22 antibody was used at 1∶200. For the immunofluorescence, the method described by Tsuneoka was used [32]. The same secondary that was used for mixed germ cell staining was applied. Following secondary antibody incubation, slides were washed in PBS and sealed using VectaMount with DAPI (Vector Laboratories). Some slides were double-stained with an acrosome marker, peanut-lectin [30].

### Live cell imaging

CHO cells transfected with full-length RC/BTB2/pEGFP-C1 plasmid were imaged with a Zeiss Cell Observer spinning disc confocal microscope equipped with a Pecon stage incubation system 16 hr after transfection. Stage and chamber temperatures were maintained at 37°C and CO_2_ levels were maintained at 5%. The cells were imaged with a 63x/1.2 n.a. PlanApo water-corrected objective lens and the 488 nm line of an Argon laser was used for illumination. A Photometrics QuantEM 512SC emCCD camera was used to collect images (with exposure times of 200 msec.) at 10 min intervals over indicated period. Axiovision software (ver. 4.8.2; Carl Zeiss Microimaging) was used to collect the image data and compile the movies.

### Electron microscope immunocytochemistry

Testis from an adult wild-type mouse was fixed and prepared for immunoelectron microscopy as previously described [33]. Sections were reacted with 13–22 antibody in a 1∶200 dilution, washed, and then incubated with anti-rabbit immunoglobulin G labeled with 10 nm gold particles.

## Results

### Tissue distribution of *Rc/btb2* mRNA

**Figure 1:**
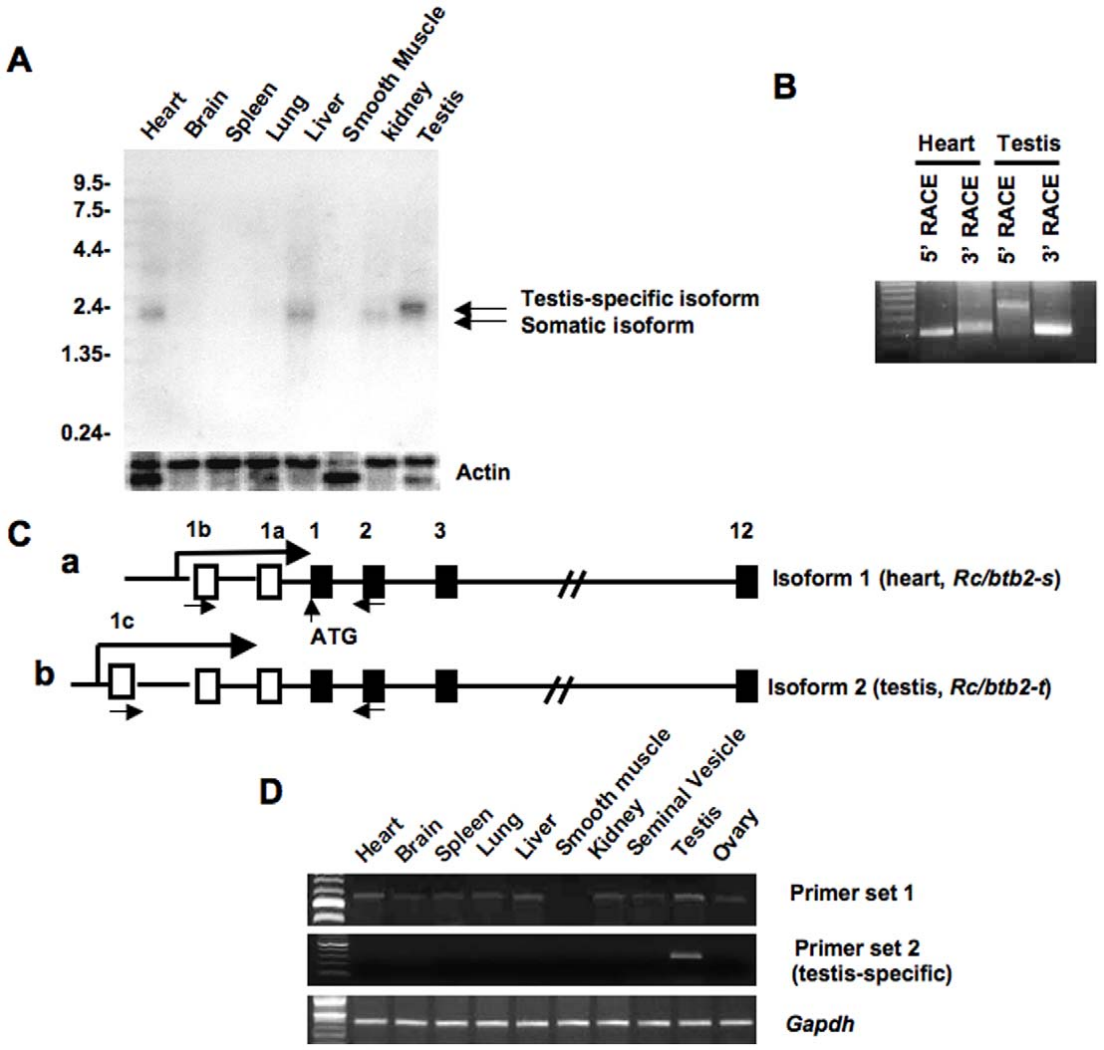
The *Rc/btb2* gene encodes two major messages. A. Analysis of *Rc/btb2* mRNA expression in the indicated tissues by Northern blot analysis. A multiple tissue blot was hybridized to a ^32^P-α-dCTP labeled *Rc/btb2* cDNA probe, the blot was exposure to an X-ray film overnight. Notice that the 2.3 kb message is present in most of the somatic tissues, the 2.5 kb message is only present in the testis. B. Identification of two transcripts (*Rc/btb2-s* and *Rc/btb2-t*) by 5′- and 3′- RACE experiments using mouse heart and testis cDNA libraries; C: Schematic representation of the two transcripts. *Rc/btb2-t* contains an extra exon (1c) compared to *Rc/btb2-s*; D: Examination of tissue distribution of the two transcripts by RT-PCR using primer sets indicated in (C). *Rc/btb2-t* is only present in the testis.

To determine the tissue distribution of *Rc/btb2* mRNA, Northern blotting was conducted. A cDNA containing the 1668 bp full-length coding region of *Rc/btb2* was labeled by ^32^P-α-dCTP, the labeled probe was hybridized to a mouse multiple tissue blot which contains mRNAs from the heart, brain, spleen, lung, liver, smooth muscle, kidney and testis. An overnight exposure revealed that *Rc/btb2* mRNA is present in the heart, liver, kidney and testis (Figure 1A). A longer exposure (4 days) showed that *Rc/btb2* mRNA is expressed in all of the tissues studied except smooth muscle (Figure S1). The message in the testis is larger in size than that seen in other tissues (Figure 1A and Figure S1). In addition, the probe also detected a 4 kb message after long exposure (Figure S1).

### The *Rc/btb2* encodes two major mRNAs

Given that the *Rc/btb2* mRNA in the testis is larger in size on Northern blotting than the mRNAs in somatic tissues, RACE experiments were conducted to amplify both 5′- and 3′- ends of *Rc/btb2* transcripts using mouse testis and heart cDNA libraries. 3′-RACE demonstrated that the PCR products amplified from the testis and heart cDNAs were same in size (Figure 1B). The DNA sequences of the two PCR products were identical. However, the 5′-RACE PCR product from the testis cDNA library was larger than that from the heart cDNA library using the same reverse primer set (Figure 1B). The 5′-RACE products were also sequenced. The full-length heart and testis cDNA sequences were then identified, and the gene structures in the two tissues are shown as Figure 1C. The heart isoform (*Rc/btb2-s,* GenBank Accession number JN086338) contains 14 exons, the first two exons (exon 1a and 1b) are not translated; the first translational starting site is in the third exon. The testis isoform (*Rc/btb2-t,* GenBank Accession number JN086339) includes 15 exons. Exons 2 to 12 are identical to those of the heart mRNA, but there is an extra non-translated exon (exon 1c) in the 5′-end of the testis transcript (Figure 1C).

To examine tissue distribution of the two transcripts further, RT-PCR was conducted with two primer sets that share the same reverse primer located within exon 3 (shown in Figure 1C), but differ in their forward primers. The forward primer of set 1 is in non-translated exon 1b, the forward primer of set 2 is in non-translated exon 1c. *Rc/btb2* message (*Rc/btb2-s*) was amplified from all the tissues except smooth muscle using primer set 1 (Figure 1D, upper panel). *Rc/btb2* message (*Rc/btb2-t*) was amplified only in the testis using primer set 2 (Figure 1D, lower panel).

### *Rc/btb2* mRNA expression in the testis during the first wave of spermatogenesis

**Figure 2:**
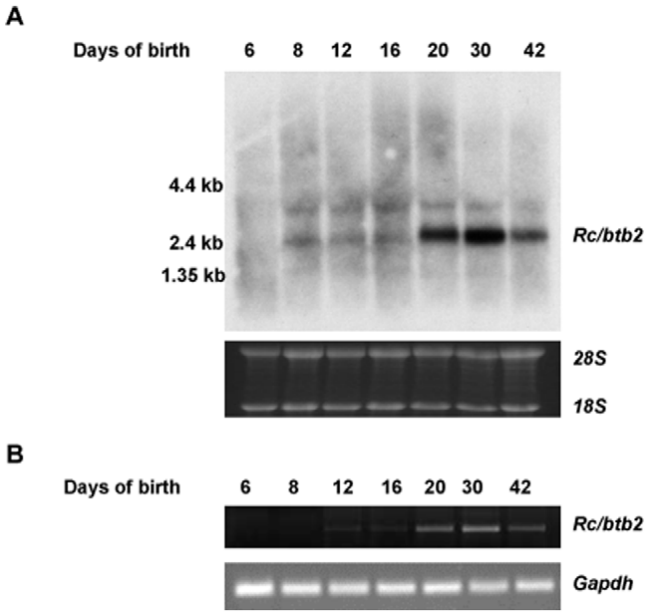
*Rc/btb2* mRNA expression in the testis during the first wave of spermatogenesis. Total testicular RNA was extracted from mouse at indicated ages, Northern blot was conducted using the same probe as described in Fig. 1A (A). RT-PCR was also performed using the same primer set that specifically amplifies *Rc/btb2-t* (B). Both Northern blot and RT-PCR analyses indicate that *Rc/btb2-t* message is significantly increased at day 20, reaches maximum level at day 30, and declines at day 42 after birth.

Expression of the unique, testis-specific isoform of the *Rc/btb2* gene was investigated in the testis during the first wave of spermatogenesis by Northern blot analysis and RT-PCR. Northern blotting demonstrated that the message was detectable from day 8 after birth, and was dramatically increased at day 20. At day 30, the message abundance was at maximal levels, but declined at 42 day after birth (Figure 2A). A similar pattern was observed by RT-PCR analysis (Figure 2B).

### The BTB domain mediates localization of the full-length protein to vesicles closely associated with the nuclear membrane in transfected CHO cells

**Figure 3:**
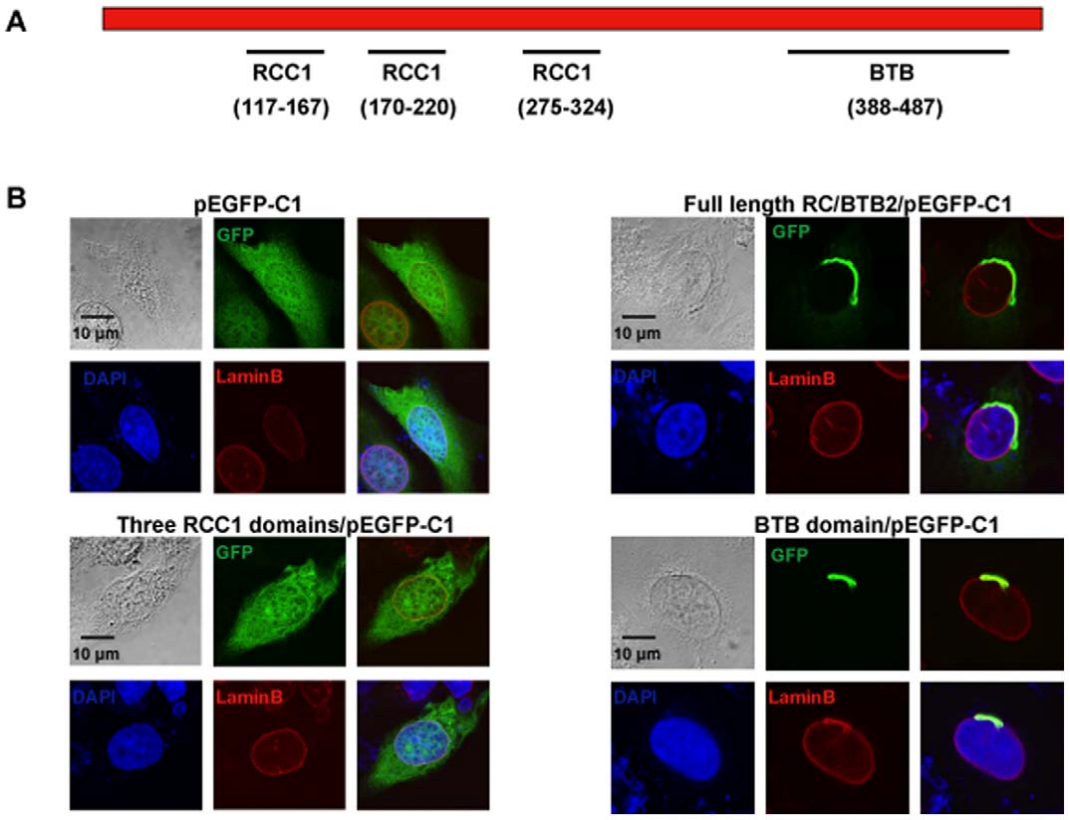
Functional analysis of domains that determine RC/BTB2 protein localization in cultured cells. A. Schematic representation of known domains of the RC/BTB2 protein as predicted by the NCBI program. The full-length RC/BTB2 protein contains three putative RCC1 domains in the N-terminus and one putative BTB domain in the C-terminus. The numbers indicate the position of these domains in the RC/BTB2 protein. B. Cellular localization of GFP tagged full-length RC/BTB2, RCC1, and BTB proteins in CHO cells. CHO cells were transfected with the indicated plasmids, 48 h after transfection, the cells were stained with an anti-Lamin B antibody, images were taken using a confocal microscope.

**Figure 4:**
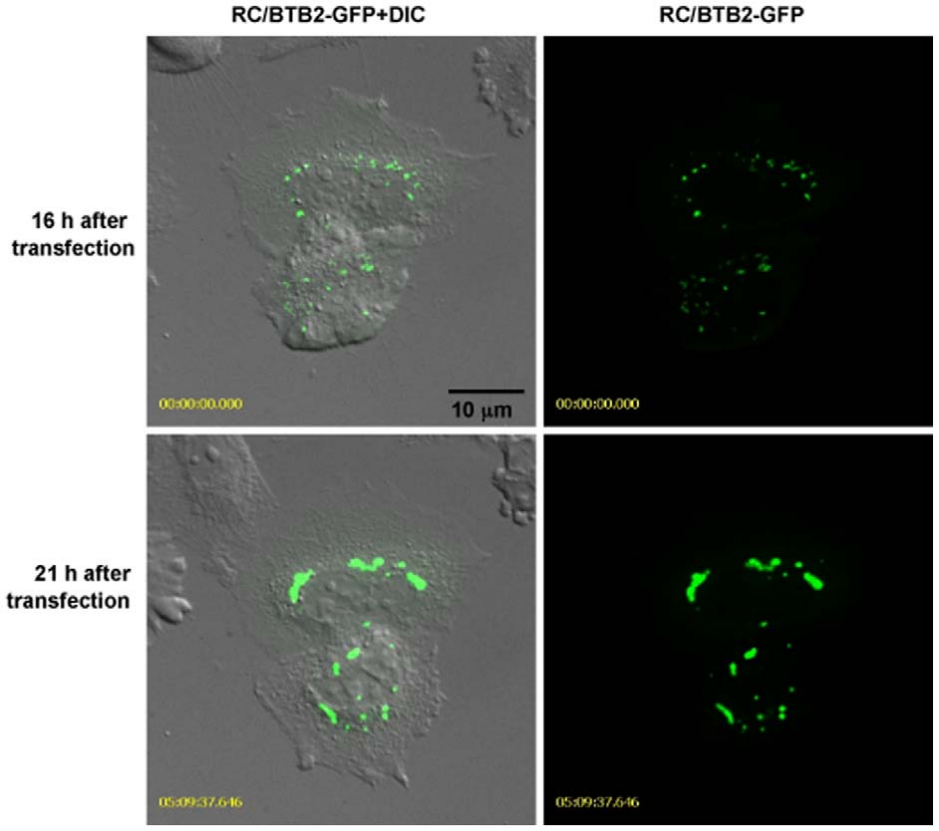
Examination of full-length GFP-tagged RC/BTB2 localization dynamically in transfected CHO cells. CHO cells were transfected by full-length RC/BTB2/pEGFP-C1 plasmid, living cell images were taken. Upper panel shows the images 16 hr after transfection, lower panel shows the images 21 hr after transfection. Notice that multiple individual granules were present in the cytoplasm 16 hr after transfection, but these granules fused together and appeared to attach to the nuclear membrane 21 hr after transfection. The dynamic process is shown in Movie S1.

To predict potential functions of this protein, conserved domains were searched using the NCBI program (http://www.ncbi.nlm.nih.gov/Structure/cdd/cdd.shtml). This analysis revealed that the full-length RC/BTB2 protein contains three conserved RCC1 domains in the N-terminus, and one BTB domain in the C-terminus (Figure 3A). Three GFP fusion constructs, which encode the full-length protein, the N-terminal RCC1 domains, and the C-terminal BTB domain, respectively, were generated. To test protein expression, the three plasmids were transfected into COS-1 cells, and Western blotting was conducted using the total cellular extracts with an anti-GFP antibody (Roche, cat: 11814460001) and the two antibodies that were raised against both ends of the full-length protein. The GFP antibody recognized the three GFP fusion proteins at the predicted sizes (Figure S2A). The N-terminal antibody recognized the 86 kDa full-length and the 64 kDa RCC1 fusion proteins (Figure S2B), and the C-terminal antibody recognized the full-length and the 48 kDa BTB fusion proteins (Figure S2C). A similar result was obtained when CHO cells were used (data not shown). Localization of the GFP-tagged proteins in transfected cells was investigated by confocal microscopy. In transfected CHO cells, the RCC1/GFP fusion protein was present throughout the whole cytoplasm, this pattern is similar to GFP alone, indicating that the RCC1 domain does not have a role in protein targeting. However, BTB/GFP and full length/GFP proteins had similar localizations, both associated with vesicular structures near the surface of the nuclear envelope (Figure 3B, Figures S3, S4). Localization of full-length GFP-tagged RC/BTB2 in CHO cells was examined dynamically by imaging living cells. Sixteen hr after transfection, the RC/BTB2-GFP fusion protein appeared as individual granules in the cytoplasm, but these granules later fused together and appeared to attach to the nuclear membrane (Figure 4, and Movie S1). The intracellular distribution of the full-length/GFP fusion protein was examined further by Western blotting analysis using isolated fractions from transfected cells. The protein was highly enriched in the insoluble S4 and P fractions, but not in the soluble S1 to S3 fractions, this pattern was similar to a 50 kDa protein that is detected by an anti-Golgi body antibody (Figure S5). These observations are consistent with the localization of GFP-tagged proteins in transfected cells, and collectively suggest that RC/BTB2 is located in vesicles, possibly Golgi elements, to which the protein is evidently directed by the BTB domain. However, because these experiments were not conducted in male germ cells, we cannot be certain that the targeting of RC/BTB2 to the acrosomal vesicles is through the BTB domain.

### RC/BTB2 tissue distribution

**Figure 5:**
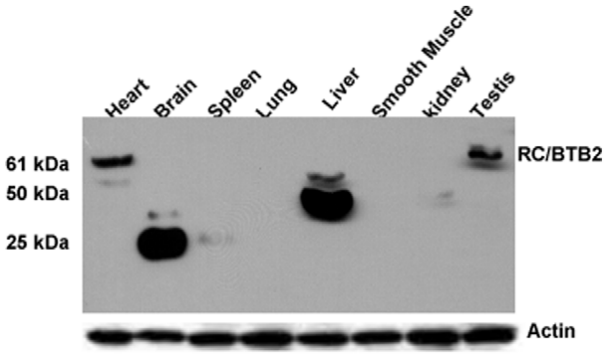
Western blot analysis of RC/BTB2 protein expression in mouse tissues. Fifty micrograms of protein from indicated mouse tissues were loaded on a SDS-PAGE gel, Western blot was conducted with an antibody generated against the N-terminus of the full-length RC/BTB2 protein. An immunoreactive protein band migrating near 61 kDa, compatible with the predicted size of full-length protein, is indicated in the heart and testis. The antibody also cross-reacts to a 25 kDa protein in the brain, and a 50 kDa protein in the liver, these might be non-specific signals or truncated proteins in these tissues, or translated proteins from other spliced isoforms from these tissues. The membrane was re-probed with anti-actin antibody as a loading control.

The tissue distribution of mouse RC/BTB2 protein was determined by Western blotting analysis using an antibody generated against the N-terminus (7610). The antibody reacted with a protein at 61 kDa, the size of the predicted full-length RC/BTB2 protein in the heart and testis. However, the antibody also cross-reacted with a 25 kDa protein in brain, and a 50 kDa protein in the liver (Figure 5).

### Localization of endogenous RC/BTB2 protein in the testis during spermatogenesis

**Figure 6:**
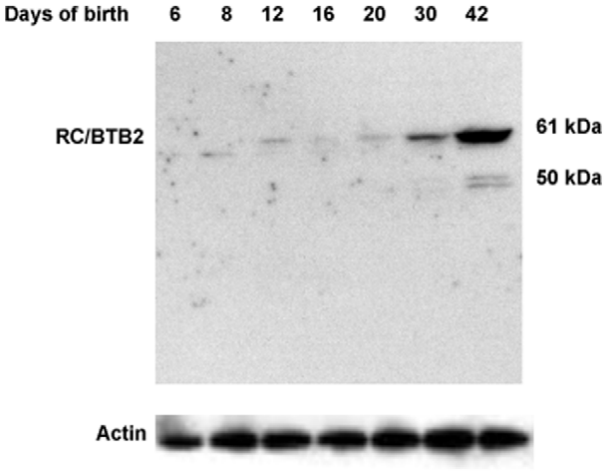
RC/BTB2 protein expression in the testis during the first wave of spermatogenesis. Testicular extracts were prepared from mice at indicated age, RC/BTB2 protein was examined by Western blot analysis using the same antibody as described in Figure 4. The 61 kDa protein is detectable from day 12 after birth, the expression level is progressively increased from day 20 after birth. A protein with lower molecular weight is also detected in the extracts from mice at day 6 and 8 after birth. The 50 kDa signal at day 42 may represent degradation product of the full-length 61 kDa protein. The membrane was re-probed with anti-actin antibody as a loading control.

To examine dynamic expression of RC/BTB2 protein in the testis during the first wave of spermatogenesis further, Western blotting was conducted using testicular extracts from mice at the indicated ages. The 61 kDa full-length protein is expressed from day 12 after birth, and the expression is significantly increased at day 20 after birth, reaching the highest level of expression at day 30 and day 42 (Figure 6). In addition, the antibody cross-reacted with a smaller protein in day 6 and day 8 testes, and a 50 kDa protein at day 30 and day 42 testes (Figure 6). These cross-reacting proteins could reflect other splice variants or post-translational modification of RC/BTB2.

**Figure 7:**
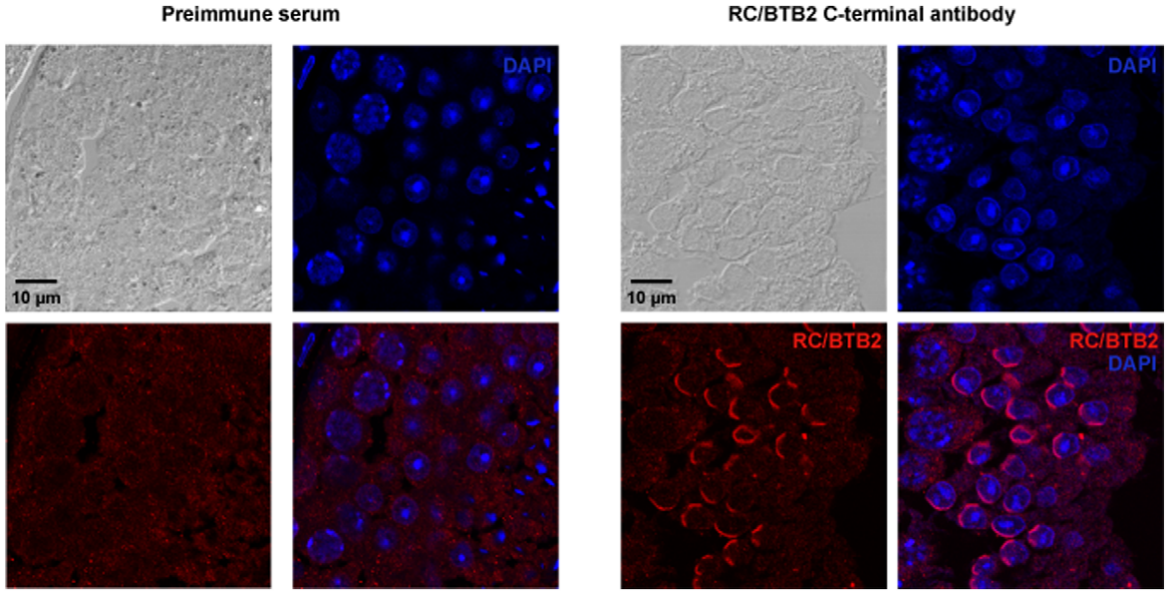
Detection of endogenous RC/BTB2 protein in mouse testicular sections. Testicular sections from adult mice were processed for immunological decoration with the 13–22 antibody. In round spermatids, labeling of a cap structure similar to the acrosomic cap was obvious. Antibody staining in red. Nuclear counterstain in blue (DAPI).

Endogenous RC/BTB2 protein localization was examined with testicular sections from adult wild-type mice using an antibody to the C-terminus. No specific staining was observed in spermatogonia. In round spermatids, the staining was largely restricted to the acrosomal cap region, and co-localized with the peanut-lectin, an acrosome marker (Figure 7, Figure S6, S7). The same localization was observed when N-terminal antibody was used (Figure S8). In some round spermatids, a strong signal was observed on the top of the acrosomal cap region (arrows in Figure S9).

**Figure 8:**
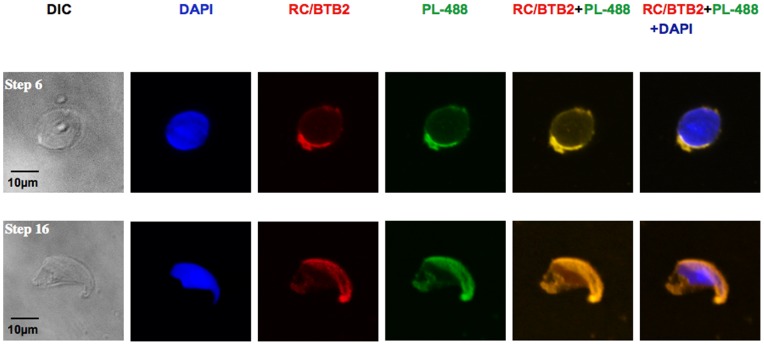
Examination of endogenous RC/BTB2 protein in suspensions of mouse testicular cells. Suspension preparations of mouse testicular cells were processed for immunological decoration with the C-terminal 13–22 antibody. Detection of the acrosomic vesicle by peanut-lectin-488 labelled (PL-488). Notice that RC/BTB2 is co-localized with PL-488 at acrosomic caps in germ cells at different stages (Step 6 spermatid in the upper panel, and step 16 spermatid in the lower panel).

**Figure 9:**
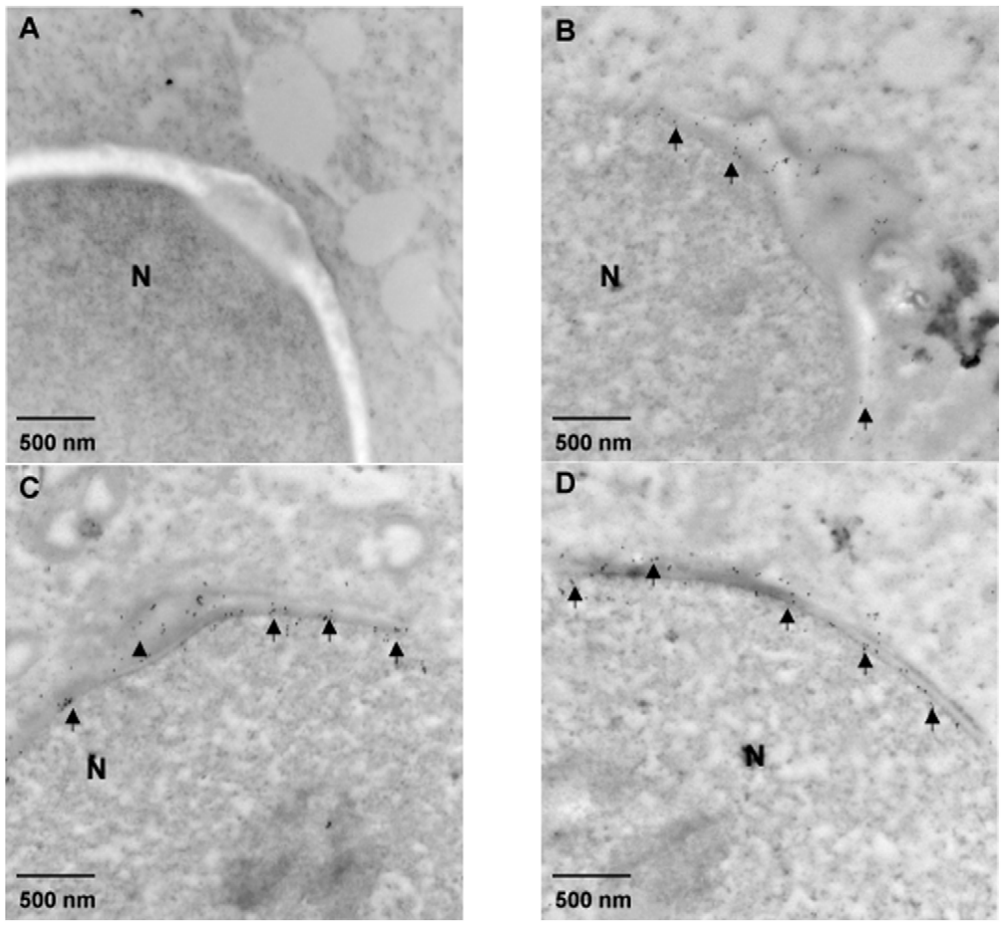
Immunoelectron microscopic localization of mouse RC/BTB2. Three representative images (B, C, and D) of spermatids from an adult wild-type mouse processed for immunoelectron microscopic detection of RC/BTB2 protein. The particles are concentrated in the acrosomic region (arrows), closely associated with nuclear membrane. The arrow heads in B and C point to developing acrosomes. A is a negative control in which the section was stained with pre-immune serum. N: Nucleus.

To analyze the sub-cellular localization of endogenous RC/BTB2 protein in germ cells further, suspension preparations of mouse testicular cells were processed, and the cells were double-stained with peanut-lectin, which recognizes the acrosome. RC/BTB2 was always associated with peanut-lectin in the acrosomal region in germ cells at different stages (Figure 8). It was also associated with sp56, another marker for the acrosome (Figure S10). The acrosomal localization was further confirmed by immunogold labeling and electron microscopy (Figure 9). The germ cells were also double-stained with an anti-α-tubulin antibody (a manchette marker). In most cells, RC/BTB2 and α-tubulin were localized at opposite poles (Figure S11). In addition, RC/BTB2 was also found in a discrete punctate distribution near the caudal region of nuclei, suggesting another function for the protein outside of the acrosome (arrows in Figure S11).

## Discussion

By yeast two-hybrid screen and RACE experiments, we identified two *Rc/btb2* transcripts, *Rc/btb2-s* and *Rc/btb2-t.* The two transcripts share the same coding sequence and encode a 551 amino acid, 61 kDa protein. The predicted protein was confirmed in the testis and heart extracts by Western blotting analysis (Figure 5).

Interestingly, in other tissues, the same antibody cross-reacted with proteins of different size, ranging from 25 to 50 kDa. Even in the day 6 and day 8 testes, a protein smaller than 61 kDa was recognized by the same antibody (Figure 6). Although Northern blot results revealed that the major *Rc/btb2* mRNA species are the same size in the somatic tissues, it is possible that other transcript isoforms that are less abundant were not detected. In Ensembl dadabase, there are 26 putative *Rc/btb2* transcripts, 20 of them are recorded as being translated into different proteins containing 5 to 551 amino acids. The proteins detected somatic tissues on Western blotting could be derived from these other *Rc/btb2* transcripts. Alternatively, they could represent post-translational modification of RC/BTB2 protein derived from the major mRNAs. These mechanisms (i.e., translation from an alternatuvely spliced minor transcript or post-translational processing) could account for differences in protein molecular weight in day 6 and day 8 testes, where somatic cells, particularly Sertoli cells, are the predominant cell types. However, we cannot exclude the possibility that these smaller proteins represent cross-reacting proteins that are distinct from those derived from the *Rc/btb2* gene.

Since RC/BTB2 was originally identified as a binding partner of SPAG16S, a male germ cell-specific protein, we were interested in characterizing it in male germ cells. During the first wave of spermatogenesis, *Rc/btb2* mRNA expression was first detected on day 8 after birth, suggesting transcription occuring with the appearance of early spermatocytes [34], and expression was dramatically increased at day 20, which corresponds to the appearance of round spermatids [34]. However, protein expression was not significantly increased until day 30 after birth, much later than the increase of mRNA expression, which would be consistent with a complement of round and elongated spermatids at the complete of the first wave of spermatogenesis [34]. This might reflect translational control of protein expression, which is characteristic of male germ cell differentiation. During spermatogenesis, many genes are transcribed at an early stage in the process, but the mRNAs are bound by RNA-binding proteins, and translation is suppressed until spermiogenesis, when the germ cells undergo dramatic remodelling with the creation of new structures, such as the acrosome [35].

In the testis, full-length RC/BTB2 expression is maintained at low levels before day 20 after birth, but after this time point, the expression level is significantly increased and maintained at high levels throughout the rest of spermatogenesis. Given that this time point (20 days) represents the transition from spermatocytes to spermatids, the expression pattern strongly suggests that the function of RC/BTB2 is related to the final phase of spermatogenesis, spermiogenesis. This notion is supported by *in vivo* localization studies in germ cells. In spermatids, the protein co-localizes with two different acrosomal markers, peanut-lectin (Figure 8) and sp56 (Figure S7, S10). The localization to acrosomal vesicles of spermatids was confirmed further by immunogold staining (Figure 9).

The acrosome is a unique organelle located over the anterior part of the sperm nucleus that is highly conserved through evolution [36]. It was initially thought to be a modified lysosome [37], but subsequent studies established that the acrosome is a Golgi-derived secretory vesicle [38], [39]. The acrosome forms during the beginning of spermiogenesis [4]. During the Golgi phase, pro-acrosomic vesicles form a single large acrosomic vesicle, which associates with the nuclear envelope via the perinuclear theca (PT). In the cap phase, the acrosomic vesicle enlarges and covers the nucleus to take the shape of a cap. The PT is subdivided into subacrosomal and postacrosomal regions [40]. The subacrosomal layer is located between the inner acrosomal membrane and the outer nuclear membrane (ONM). Posterior to the acrosome is the postacrosomal sheath of the PT, which is sandwiched between the plasmalemma and the ONM. The two parts of the PT have different functions. The subacrosomal layer is involved in acrosome biogenesis. Docking of the acrosome to the nucleus is mediated by the acroplaxome, a cytoskeletal plate consisting of F-actin, SAK57 and myosin Va [41]–[43]. The acroplaxome nucleates an F-actin–keratin-containing assembly with the purpose of stabilizing and anchoring the developing acrosome during spermatid nuclear elongation [44].

Although the Golgi origin of the acrosome is accepted, Berruti and Paiardi recently revisited the notion that the acrosome is a novel lysosome-related organelle (LRO) [36]. LROs have functional and dynamic stages of maturation as indicated by the involvement of many Rab family proteins, small GTPases critical for vesicle fusion and transport [45]–[47]. The conserved RCC1 domains, and the images from living cells transfected with GFP-tagged RC/BTB2 suggest that this protein might play a role in vesicle fusion and the transport of material inside of the acrosome during acrosome formation. Since full length RC/BTB2 protein has three RCC1 domains in the N-terminus, it is possible that the protein binds to Ran through one RCC1 domain, but also binds to other proteins, such as Rab through the other two RCC1 domains. The characteristics of RC/BTB2 are consistent with the notion that the acrosome is a LRO, and we are attempting to other binding partners in yeast two-hybrid screening and co-immunoprecipitation experiments.

Many proteins within the acrosomal vesicles are glycosylated [48]. However, RC/BTB2 is evidently not subjected to major post-translational modification, since the calculated size of the protein in the Western blot analyses (61 KDa) is consistent with the expected molecular weight derived from the amino acid sequence (60.15 KDa). When searching for conserved domains of RC/BTB2 protein, no signal peptide was identified that could account for targeting the protein into Golgi elements or secretory vesicles. Besides the three RCC1 domains, RC/BTB2 protein has one conserved BTB domain in the C-terminus, and this domain appears to target RC/BTB2 protein localization to vesicles near the surface of the nuclear envelope of cultured cells (Figure 3, Figure S3), localization of RC/BTB2 protein in spermatids may also be directly or indirectly determined by this domain.

Interestingly, mouse HERC4, an ubiquitin ligase, also contains RCC1 domain in the N-terminus. This protein is highly expressed in the testis, and has a developmental pattern of expression almost identical to RC/BTB2 [49]. Amino acid alignment analysis of the two proteins revealed that the amino acid identity was 32.1% in 274 overlapping amino acid. This degree of similarity is not likely to have influenced our immunocytochemistry and Western blot studies as a result of antibody cross-reactivity.

In summary, we characterized mouse RC/BTB2, a previously unstudied protein. Its conserved domains, dynamic expression pattern during spermatogenesis, and cellular localization strongly suggest that the protein plays a role in the acrosome during spermiogenesis.

## Supporting Information

Figure S1 **The** ***Rc/btb2*** **gene encodes two major messages.** Analysis of *Rc/btb2* mRNA expression in the indicated tissues by Northern blot analysis. A multiple tissue blot was hybridized to a ^32^P-α-dCTP labeled *Rc/btb2* cDNA probe, the blot was exposure to an X-ray film for four days.
(TIF)

Figure S2 **Analysis of GFP fusion protein expression in transfected COS-1 cells by Western blot.** COS-1 cells were transfected with the full-length RC/BTB2-pEGFP-C1, RCC1-pEGFP-C1 or BTB-pEGFP-C1 vectors. Forty-eight h after transfection, total cell lysates were prepared and Western blots were performed with an anti-GFP antibody (A), N-terminal 7610 antibody (B), and C-terminal 13–22 antibody (C). The membranes were re-probed with anti-actin antibody as a loading control.
(TIF)

Figure S3 **Localization of GFP tagged full-length RC/BTB2 protein in CHO cells.**CHO cells were transfected with RC/BTB2/pEGFP-C1 plasmid, 48 h after transfection, the cells were stained with an anti-Lamin B antibody, images were taken using confocal laser-scanning microscopy.
(TIF)

Figure S4 **Localization of GFP tagged BTB domain in CHO cells**. CHO cells were transfected with BTB/pEGFP-C1 plasmid, 48 h after transfection, images were taken using a confocal laser-scanning microscopy.
(TIF)

Figure S5 **Intracellular distribution of full-length RC/BTB2-GFP fusion protein in transfected mammalian cells**. COS-1 cells were transfected with RC/BTB2-pEGFP-C1 plasmid, and subjected to subsequent biochemical fractionation. Following centrifugation, soluble and insoluble fractions were produced. The soluble fractions are S1–S4. Cytoplasmic proteins soluble in hypotonic buffer are found in S1. Proteins soluble in 50 mM NaCl, 1% Triton X-100 are found in S2, proteins soluble in 500 mM NaCl, 1% Triton X-100 are found in S3 and proteins in 7M urea found in S4. Proteins not even soluble in 7M urea are found in the pellet P. proteins were separated on SDS-PAGE gels, transferred to PVDF membranes, and probed with indicated antibodies. The 50 kDa lower band may be a proteolytically processed form of the 97 kDa protein.
(TIF)

Figure S6 **Detection of endogenous RC/BTB2 protein in mouse testicular sections.** Testicular sections from adult mice were processed for immunological decoration with the 13–22 antibody. In round spermatids labeling of a cap structure similar to the acrosomic cap was obvious. Antibody staining in red. Nuclear counterstain in blue (DAPI).
(TIF)

Figure S7 **RC/BTB2 co-localizes with peanut lectin in mouse testicular sections.** Testicular sections from adult mice were processed for immunological decoration with the 13–22 antibody and peanut-lectin-488 labelled (PL-488). Notice that RC/BTB2 is co-localized with PL-488 at acrosomic caps. Notice that a ring-like structure, possibly the Golgi body, was also stained in earlier germ cells (white arrows).
(TIF)

Figure S8 **Detection of endogenous RC/BTB2 protein in mouse testicular cryosections using the antibody against N-terminus.** Testicular cryosections from adult mice were processed for immunological decoration with pre-immune serum (left) and the 7610 antibody (right). In round spermatids, the 7610 antibody detected antigen in a cap-like structure. Antibody staining in green with 488-conjugated anti-rabbit secondary antibody. Nuclear counterstain in blue (DAPI).
(TIF)

Figure S9 **RC/BTB2 concentrates in the cap region in some round spermatids.** Images showing that RC/BTB2 protein concentrates in the cap region (arrows) in some round spermatids in testicular sections from adult mice.
(TIF)

Figure S10 **RC/BTB2 co-localizes with sp56 in spermatids.** Suspension preparations of mouse testicular cells were processed for immunological decoration with the C-terminal 13-22 antibody. The acrosomic vesicle was detected by anti-sp56 antibody. Notice that RC/BTB2 is co-localized with sp56 in acrosomic caps.
(TIF)

Figure S11 **Opposite localization of RC/BTB2 and α-tubulin in spermatids.** Suspension preparations of mouse testicular cells were processed for immunological decoration with C terminal 13–22 antibody and anti-α-tubulin antibody. RC/BTB2 was stained in red with Cyc3 labeled anti-rabbit secondary antibody, α-tubulin was stained in green with 488-conjugated anti-mouse secondary antibody. The arrows point to centriol like localization of RC/BTB2 protein.
(TIF)

Table S1 **Primers used in this study.**
(DOC)

Movie S1 **Live cell imaging of CHO cells transfected with RC/BTB2/pEGFP-C1 plasmid.** CHO cells transfected with full-length RC/BTB2/pEGFP-C1 plasmid were imaged with a Zeiss Cell Observer spinning disc confocal microscope equipped with a Pecon stage incubation system 16 hr after transfection. Note that the RC/BTB2-GFP fusion protein appeared as individual granules in the cytoplasm, but these granules later fused together and appeared to attach to the nuclear membrane.
(AVI)
