# Supplementary figures and images for: Mouse RC/BTB2, a Member of the RCC1 Superfamily, Localizes to Spermatid Acrosomal Vesicles
Source: PLoS One. 2012 Jun 29;7(6):e39846. doi: 10.1371/journal.pone.0039846 (PMC3387240; doi:10.1371/journal.pone.0039846)

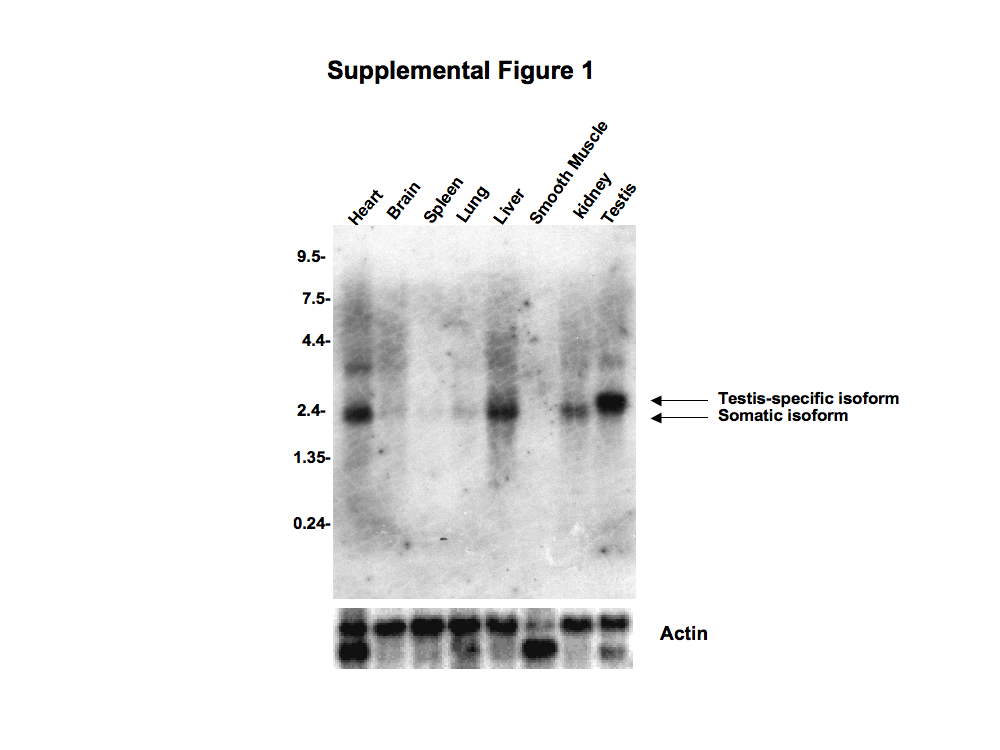

Supplement: Figure S1 — The Rc/btb2 gene encodes two major messages. Analysis of Rc/btb2 mRNA expression in the indicated tissues by Northern blot analysis. A multiple tissue blot was hybridized to a 32P-α-dCTP labeled Rc/btb2 cDNA probe, the blot was exposure to an X-ray film for four days. (TIF) [file pone.0039846.s001.tif]

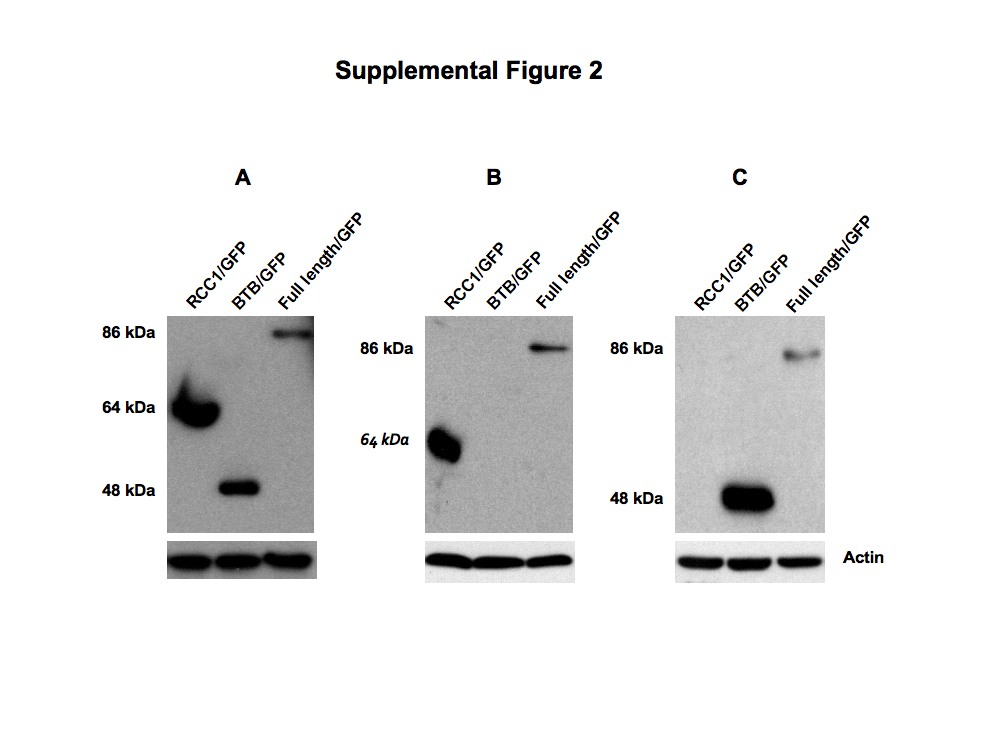

Supplement: Figure S2 — Analysis of GFP fusion protein expression in transfected COS-1 cells by Western blot. COS-1 cells were transfected with the full-length RC/BTB2-pEGFP-C1, RCC1-pEGFP-C1 or BTB-pEGFP-C1 vectors. Forty-eight h after transfection, total cell lysates were prepared and Western blots were performed with an anti-GFP antibody (A), N-terminal 7610 antibody (B), and C-terminal 13–22 antibody (C). The membranes were re-probed with anti-actin antibody as a loading control. (TIF) [file pone.0039846.s002.tif]

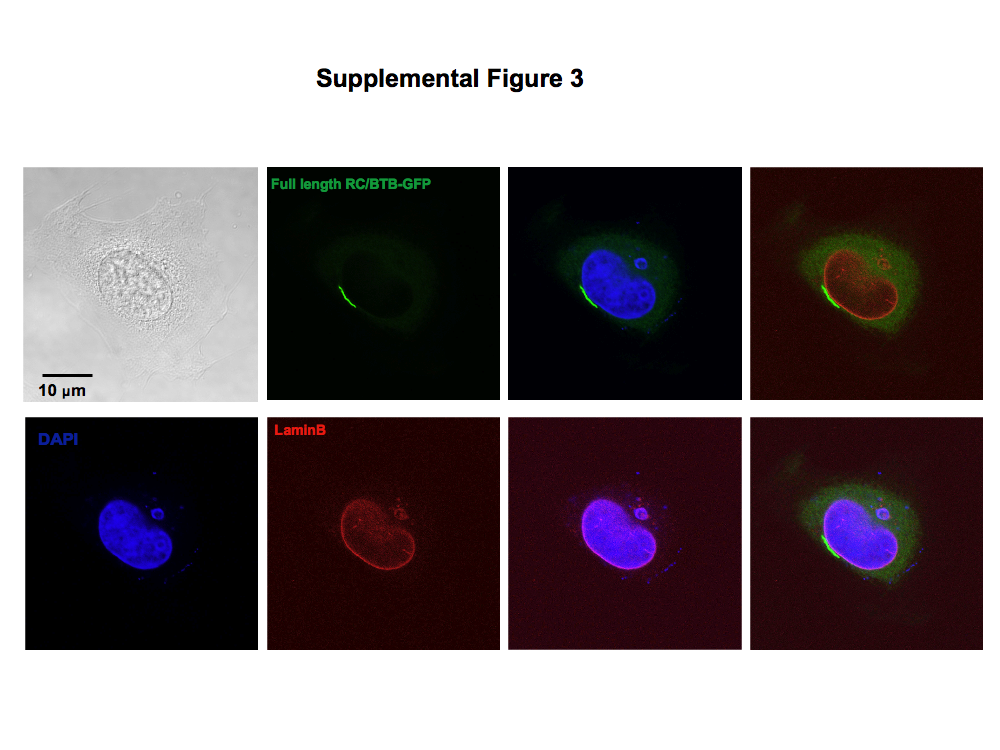

Supplement: Figure S3 — Localization of GFP tagged full-length RC/BTB2 protein in CHO cells.CHO cells were transfected with RC/BTB2/pEGFP-C1 plasmid, 48 h after transfection, the cells were stained with an anti-Lamin B antibody, images were taken using confocal laser-scanning microscopy. (TIF) [file pone.0039846.s003.tif]

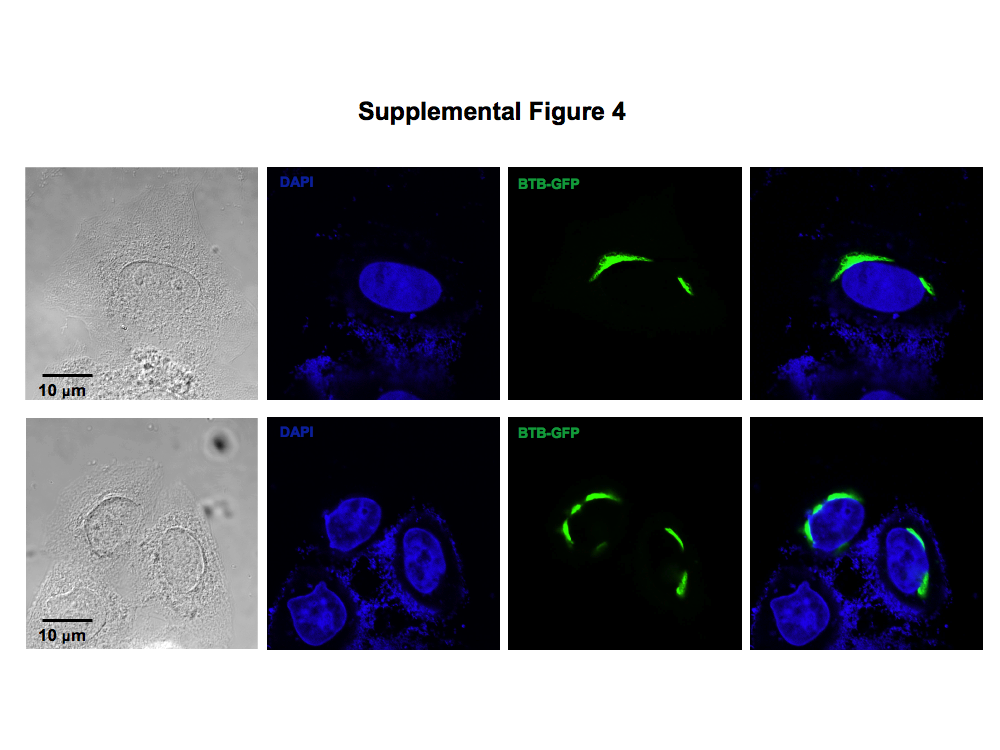

Supplement: Figure S4 — Localization of GFP tagged BTB domain in CHO cells. CHO cells were transfected with BTB/pEGFP-C1 plasmid, 48 h after transfection, images were taken using a confocal laser-scanning microscopy. (TIF) [file pone.0039846.s004.tif]

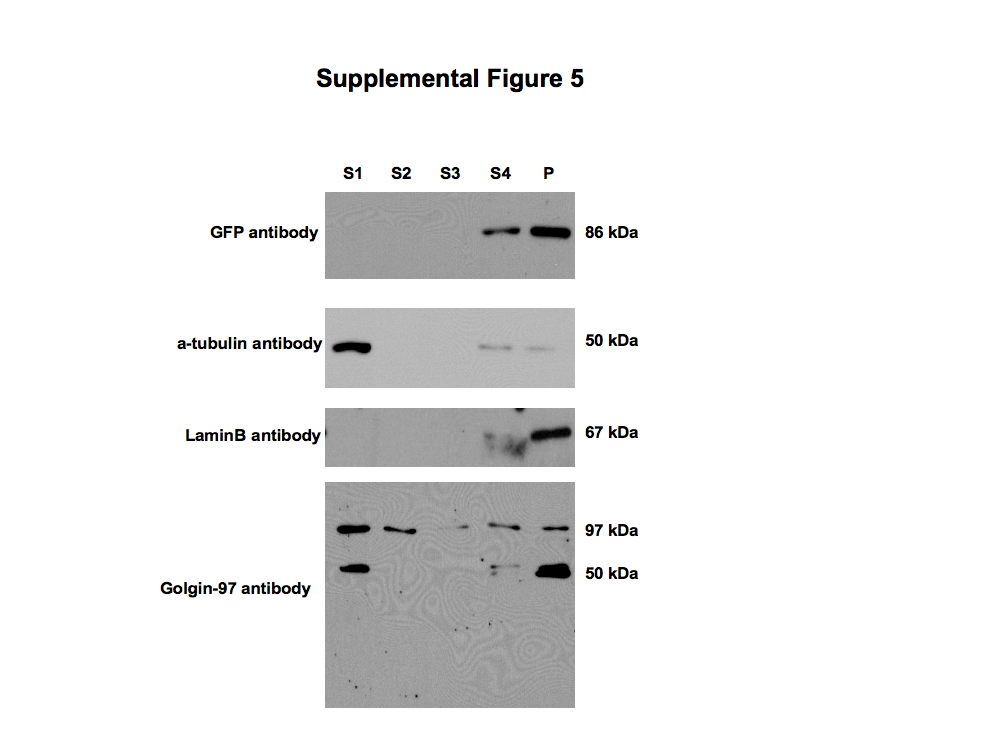

Supplement: Figure S5 — Intracellular distribution of full-length RC/BTB2-GFP fusion protein in transfected mammalian cells. COS-1 cells were transfected with RC/BTB2-pEGFP-C1 plasmid, and subjected to subsequent biochemical fractionation. Following centrifugation, soluble and insoluble fractions were produced. The soluble fractions are S1–S4. Cytoplasmic proteins soluble in hypotonic buffer are found in S1. Proteins soluble in 50 mM NaCl, 1% Triton X-100 are found in S2, proteins soluble in 500 mM NaCl, 1% Triton X-100 are found in S3 and proteins in 7M urea found in S4. Proteins not even soluble in 7M urea are found in the pellet P. proteins were separated on SDS-PAGE gels, transferred to PVDF membranes, and probed with indicated antibodies. The 50 kDa lower band may be a proteolytically processed form of the 97 kDa protein. (TIF) [file pone.0039846.s005.tif]

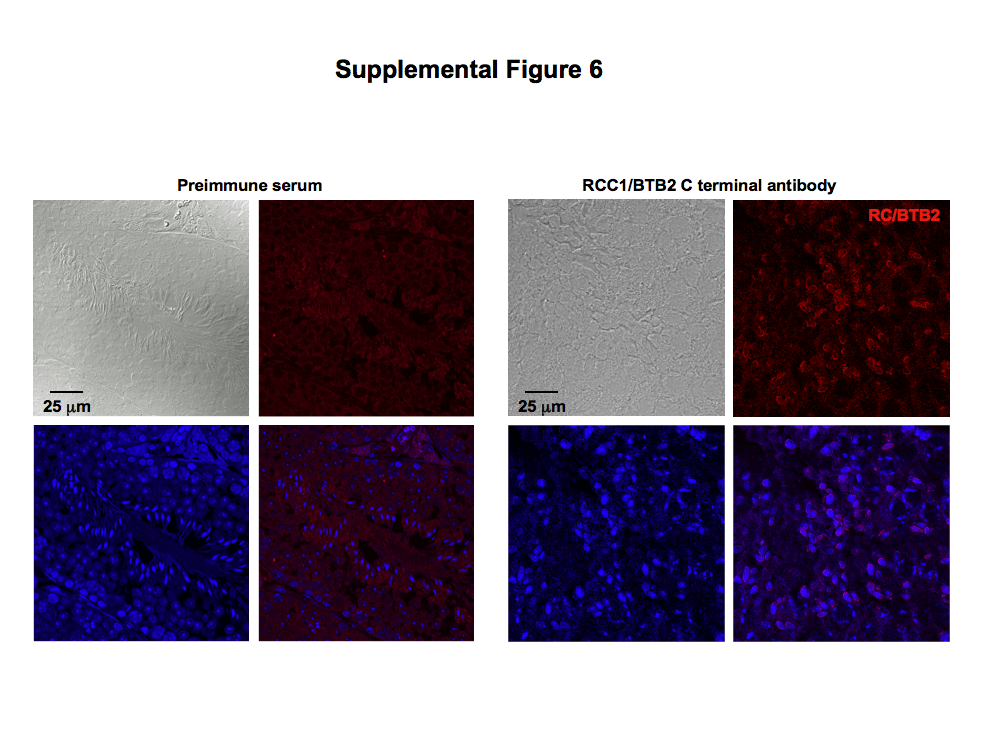

Supplement: Figure S6 — Detection of endogenous RC/BTB2 protein in mouse testicular sections. Testicular sections from adult mice were processed for immunological decoration with the 13–22 antibody. In round spermatids labeling of a cap structure similar to the acrosomic cap was obvious. Antibody staining in red. Nuclear counterstain in blue (DAPI). (TIF) [file pone.0039846.s006.tif]

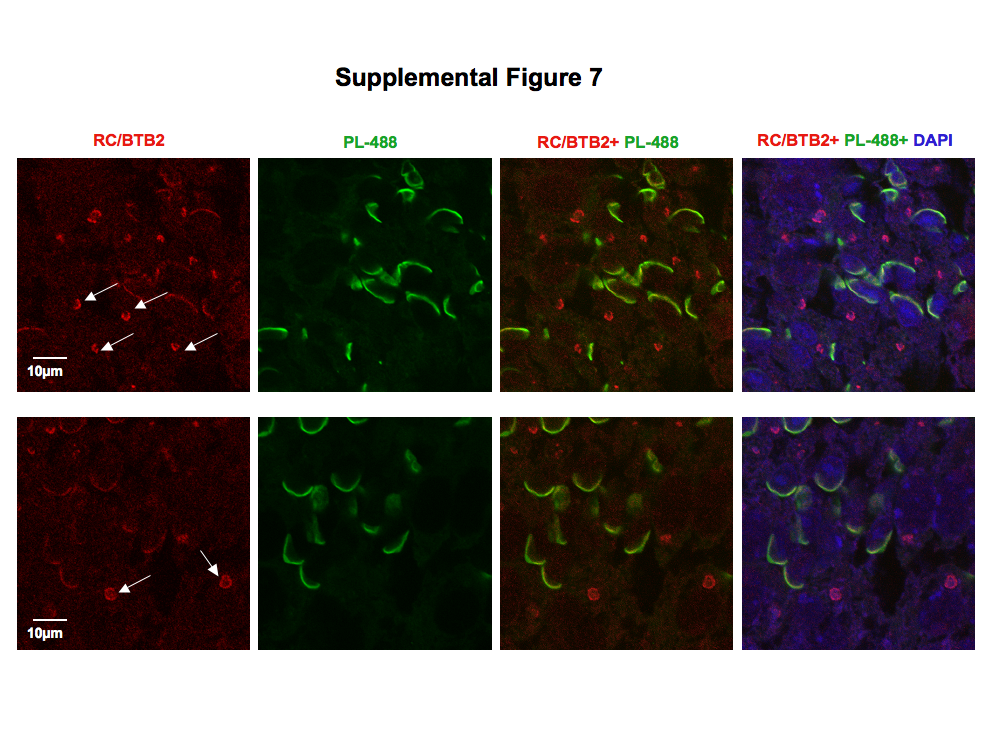

Supplement: Figure S7 — RC/BTB2 co-localizes with peanut lectin in mouse testicular sections. Testicular sections from adult mice were processed for immunological decoration with the 13–22 antibody and peanut-lectin-488 labelled (PL-488). Notice that RC/BTB2 is co-localized with PL-488 at acrosomic caps. Notice that a ring-like structure, possibly the Golgi body, was also stained in earlier germ cells (white arrows). (TIF) [file pone.0039846.s007.tif]

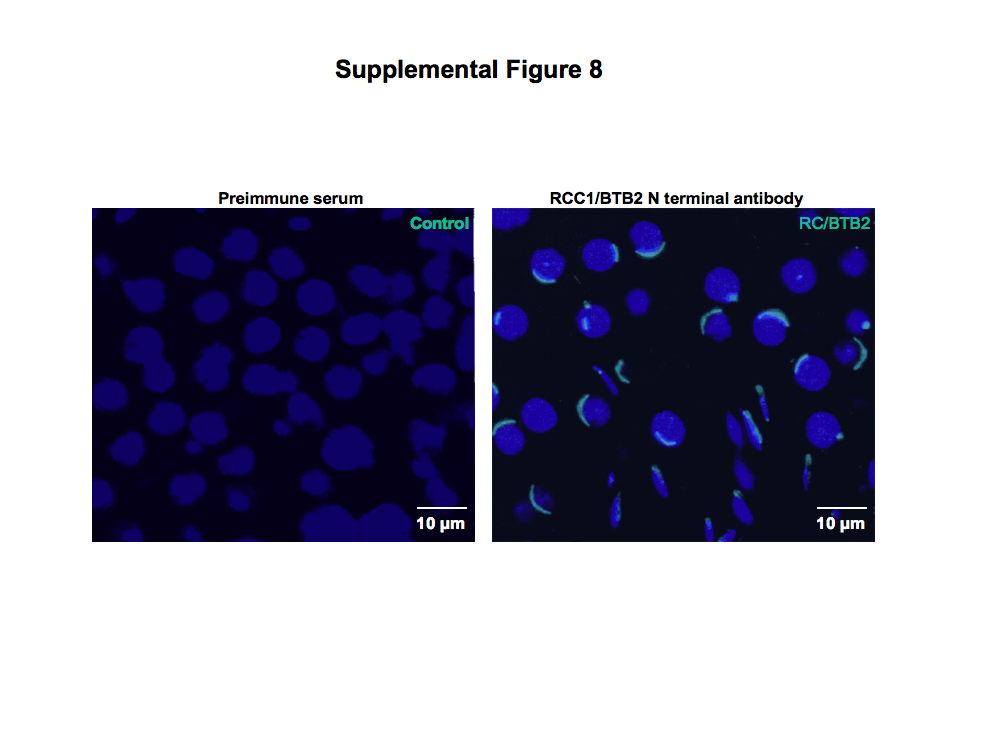

Supplement: Figure S8 — Detection of endogenous RC/BTB2 protein in mouse testicular cryosections using the antibody against N-terminus. Testicular cryosections from adult mice were processed for immunological decoration with pre-immune serum (left) and the 7610 antibody (right). In round spermatids, the 7610 antibody detected antigen in a cap-like structure. Antibody staining in green with 488-conjugated anti-rabbit secondary antibody. Nuclear counterstain in blue (DAPI). (TIF) [file pone.0039846.s008.tif]

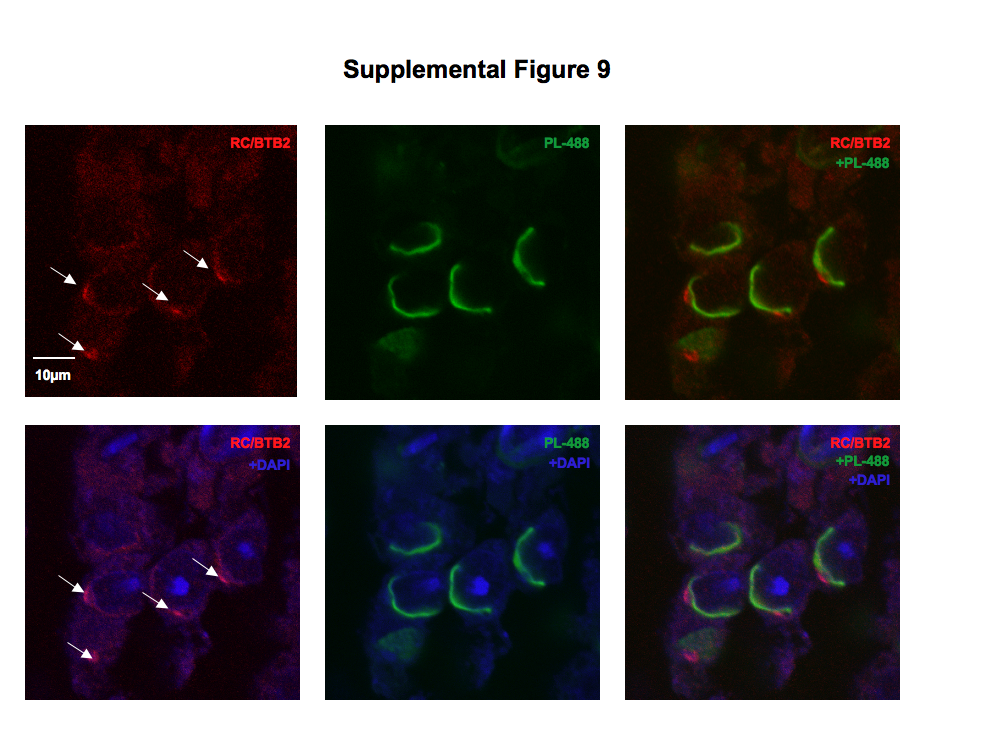

Supplement: Figure S9 — RC/BTB2 concentrates in the cap region in some round spermatids. Images showing that RC/BTB2 protein concentrates in the cap region (arrows) in some round spermatids in testicular sections from adult mice. (TIF) [file pone.0039846.s009.tif]

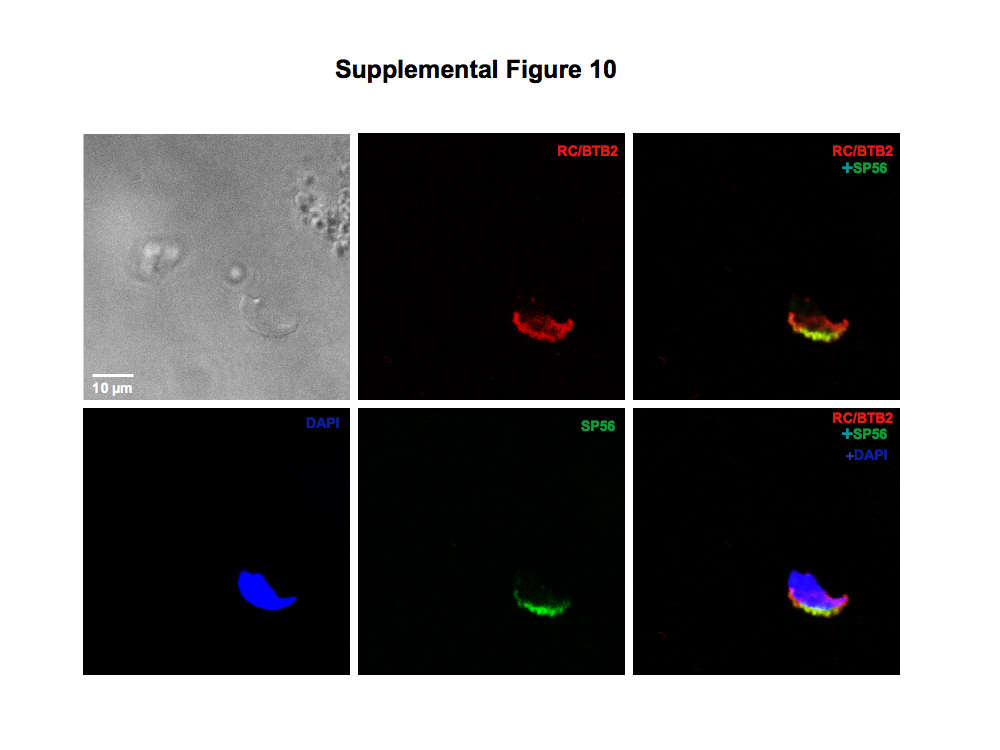

Supplement: Figure S10 — RC/BTB2 co-localizes with sp56 in spermatids. Suspension preparations of mouse testicular cells were processed for immunological decoration with the C-terminal 13-22 antibody. The acrosomic vesicle was detected by anti-sp56 antibody. Notice that RC/BTB2 is co-localized with sp56 in acrosomic caps. (TIF) [file pone.0039846.s010.tif]

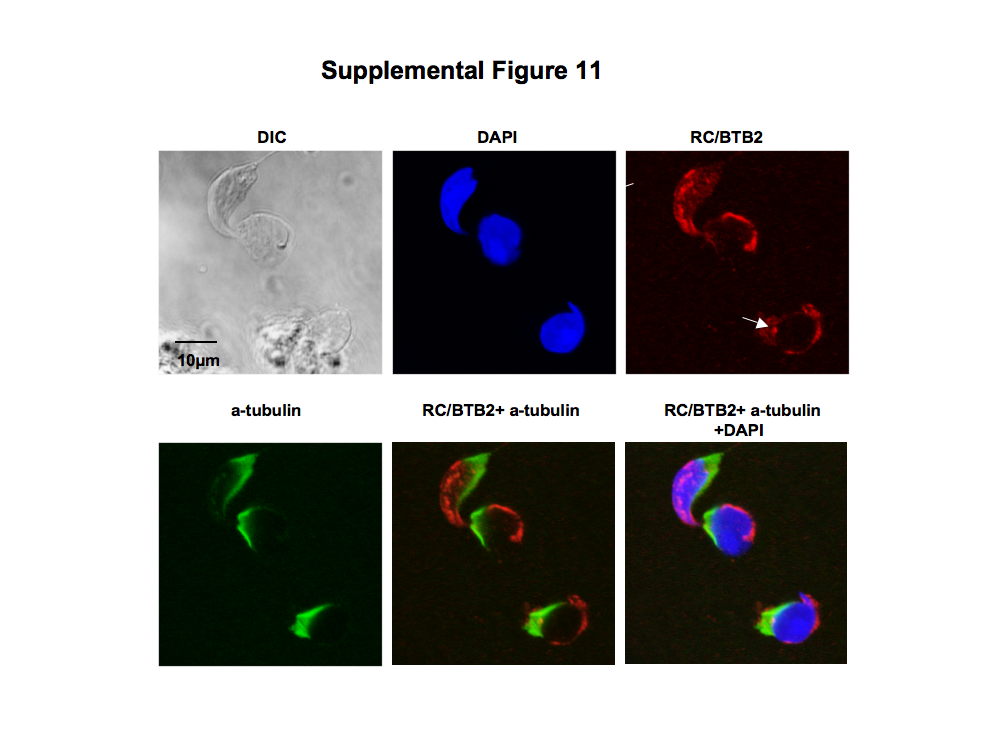

Supplement: Figure S11 — Opposite localization of RC/BTB2 and α-tubulin in spermatids. Suspension preparations of mouse testicular cells were processed for immunological decoration with C terminal 13–22 antibody and anti-α-tubulin antibody. RC/BTB2 was stained in red with Cyc3 labeled anti-rabbit secondary antibody, α-tubulin was stained in green with 488-conjugated anti-mouse secondary antibody. The arrows point to centriol like localization of RC/BTB2 protein. (TIF) [file pone.0039846.s011.tif]
