# Supplementary material for: Mouse RC/BTB2, a Member of the RCC1 Superfamily, Localizes to Spermatid Acrosomal Vesicles
Source: PLoS One. 2012 Jun 29;7(6):e39846. doi: 10.1371/journal.pone.0039846 (PMC3387240; doi:10.1371/journal.pone.0039846)
Supplement: Table S1 — Primers used in this study. (DOC) [file pone.0039846.s012.doc]

Table S1: primers used in this study.

| Primer names | Primer sequences |
| --- | --- |
| *Rc/btb2* 5’-RACE primer | 5’-cttgccactttctccatagaaacc-3’ |
| *Rc/btb2* 3’-RACE primer | 5’-gctctctgctgctgtcaagtatg-3’ |
| *Rc/btb2* cDNA probe forward | 5’-catatggaagaagaagtgcctggt-3’ |
| *Rc/btb*2 cDNA probe reverse | 5’-ggatcagttcttaaaggctccaac-3’ |
| *Rc/btb2-s* RT-PCR forward | 5’-ctggaagacagctttacaagaatc-3’ |
| *Rc/btb2-s(t)* RT-PCR reverse | 5’-ttccatatggatcagtcctgtcag-3’ |
| *Rc/btb2-t* RT-PCR forward | 5’-gtggaggagtttagaggtgaaaaac-3’ |
| N-RC/BTB2/PET28A forward | 5’-gctagcatggaagaagaagtgcct-3’ (NheI) |
| N-RC/BTB2/PET28A reverse | 5’-gaattcaccttgtgtcttggcagc-3’ (EcoRI) |
| C-RC/BTB2/PET28A forward | 5’-gctagcgaacctgacgaccacctcact-3’ (NheI) |
| C-RC/BTB2/PET28A reverse | 5’-gaattcgttcttaaaggctccaactct-3’ (EcoRI) |
| Full length RC/BTB2/pEGFP-C1 forward | 5’-gaattctctgatccatatggaagaagaagtg-3’ (EcoRI) |
| Full length RC/BTB2/pEGFP-C1 reverse | 5’-ggatccggatcagttcttaaaggctccaac-3’ (BamHI) |
| RCC1/pEGFP-C1 reverse * | 5’-ggatccgtgagtgtggtgtggaaggatcac-3’ (BamHI) |
| BTB/ pEGFP-C1 forward** | 5’-gaattctgtgatccttccacaccacactcac-3’ (EcoRI) |

* The forward primer is the same as used for the full-length construct; **The reverse primer is the same as used for the full length construct.
